# Effects of reflection and immediate feedback to improve clinical reasoning of medical students in the assessment of dermatologic conditions: a randomised controlled trial

**DOI:** 10.1186/s12909-020-02063-y

**Published:** 2020-05-08

**Authors:** Sungjun Choi, Sohee Oh, Dong Hun Lee, Hyun-Sun Yoon

**Affiliations:** 1 Department of Dermatology, Seoul National University Hospital, Seoul, South Korea; 2 Department of Dermatology, SMG-SNU Boramae Medical Center, 20, Boramae-ro 5-gil, Dongjak-gu, Seoul, 07061 South Korea; 3 Department of Biostatistics, SMG-SNU Boramae Medical Center, Seoul, South Korea; 4 Department of Dermatology, Seoul National University College of Medicine, Seoul, South Korea

**Keywords:** Clinical reasoning, Medication education, Dermatology elective, Reflection, Feedback

## Abstract

**Background:**

There are few studies that directly compared different interventions to improve medical students’ clinical reasoning for dermatologic conditions.

**Objective:**

To investigate the effectiveness of adding practice with reflection and immediate feedback on traditional dermatology electives in improving medical students’ ability in evaluating skin lesions.

**Methods:**

The participants were fourth-year medical students of Seoul National University College of Medicine, Korea, who were enrolled to take a 2-week dermatology elective course (*n* = 87). Students were assigned to one of the three educational interventions: 2-h training involving 10 written clinical cases (experimental); 1-h lecture and 1-h outpatient clinic (lecture); and 2-h outpatient clinic (no intervention). Before and at the end of rotation, diagnostic accuracy was estimated using 20 written clinical cases with photographs (10 novel cases presented in diagnostic training [training set], 10 cases with diagnoses not included in training [control set]).

**Results:**

There was a significant interaction effect of intervention×set×time. A post hoc analysis indicated that the students in the experimental group outperformed students in the other two groups only in the training set of the final tests; after completing the 2-week rotation, for the training set, the mean score was higher in the experimental group (7.5 ± 1.3) than in the lecture (5.7 ± 1.6) and no intervention (5.6 ± 1.3) groups, producing an effect size of 1.2 standard deviation (SD) and 1.5 SD, respectively.

**Conclusion:**

Practicing written clinical cases with reflection and feedback is superior to a lecture-based approach and yields additional benefits to a dermatology elective, thereby enhancing medical students’ ability to accurately diagnose skin lesions.

**Trial registration:**

ClinicalTrials.gov, NCT03472001. Registered 21 March 2018.

## Introduction

Primary care physicians are often expected to make a clinical decision about dermatologic conditions because of a paucity of dermatologists who could accurately diagnose skin lesions including melanoma [1]. In fact, the majority of physician-detected melanomas are discovered by primary care physicians [2]. However, medical professionals other than dermatologists may not be specifically trained to diagnose and treat skin diseases [1] .

Furthermore, the initial symptoms of certain dermatologic conditions mimic those of other organ diseases. For example, herpes zoster could be misdiagnosed as cholecystitis, angina, or stroke when a rash is subtle or preceded by pain [3]. If primary care physicians are not adept at diagnosing certain skin diseases that they could frequently encounter, then opportunities for early diagnosis and appropriate treatment could be missed [1].

Artificial intelligence (AI) diagnostic tools have been developed [4], and even the United States Food and Drug Administration recently permitted marketing of AI-based device to detect diabetes-related eye problem [5]. However, clinical reasoning of human doctors is still needed for a while. In particular, the spectrum of dermatologic diseases is very wide, and many dermatologic conditions have not been standardised. Therefore, a higher level of clinical competence may be required from specialists to be able to use the diagnostic algorithm tool properly.

To develop diagnostic competence, clinicians should gain experience in a variety of clinical problems and build their illness scripts of diseases [6]. Physicians other than dermatologists need to develop illness scripts of dermatologic diseases, especially when they are enrolled in medical schools. However, not all medical students have dermatology electives, and even medical students who have completed a dermatology elective can only experience a limited number of clinical cases [7–9].

Therefore, more effective strategies are needed to enhance medical students’ clinical reasoning in the assessment of dermatologic conditions [1]. There are many theory-based approaches designed to improve clinical reasoning [6, 10–13], and feedback and reflection are two basic teaching methods used in clinical settings [14]. However, most previous studies only compared the efficacy between an educational intervention and no intervention in improving clinical reasoning, rather than comparing different educational interventions [15]. We hypothesised that adding practice with reflection and immediate feedback (experimental intervention) in traditional dermatology electives would improve the medical students’ ability to evaluate skin lesions, in comparison with a lecture-based approach and dermatology elective alone. We also hypothesised that experimental intervention selectively improved the diagnostic accuracy for the diseases presented in the training set. To prove our hypotheses, we compared practicing written clinical cases with reflection and immediate feedback with two other control interventions (lectures and no intervention); we also compared the diagnostic accuracy in the training and the control sets.

## Methods

### Study design and participants

This single centre, unblinded, randomised controlled trial included fourth-year medical students of Seoul National University College of Medicine (SNUCM), Korea, who were enrolled to take a 2-week dermatology elective course in 2018. A total of 87 medical students applied to take the dermatology electives.

At SNUCM, all fourth-year medical students take 10 h of lecture-based dermatology classes and take written tests in February before enrolling in the dermatology electives. Subsequently, they apply to undergo rotations in six clinical departments and attend 2-week rotations in each department for a total of 12 weeks from March to June. Each dermatology rotation includes 14 to 15 students. Students attend outpatient clinics, grand rounds, and medical didactics at three different hospitals (6 days in Seoul National University Hospital, 2 days in Seoul Metropolitan Government-Seoul National University [SMG-SNU] Boramae Medical Center, and 2 days in Seoul National University Bundang Hospital).

### Randomisation and blinding

**Fig. 1:**
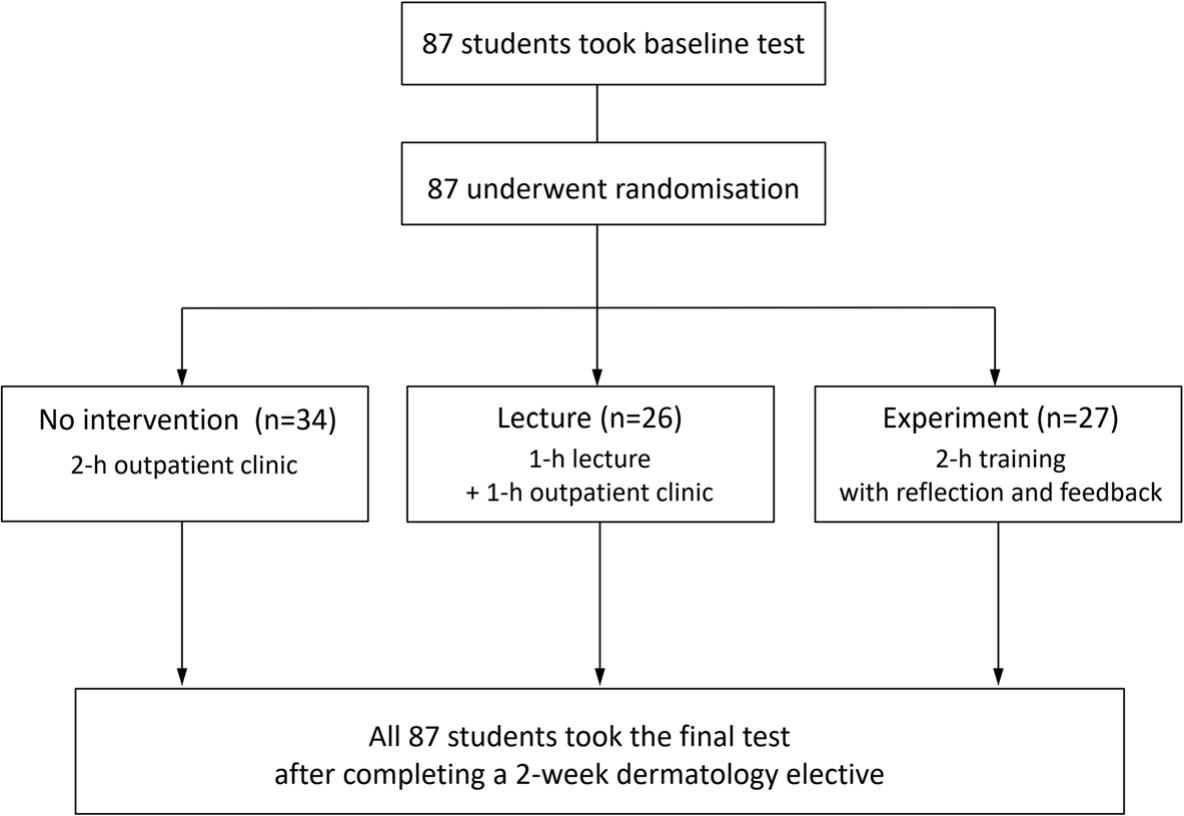
Flowchart showing randomisation of students and progress in the study

Students were randomly assigned to the experimental, lecture, and no intervention groups (3:3:4) by drawing numbered folded notes at random from a bag on the first day of their dermatology elective course. One the second day of the 2-week elective course, students underwent three different educational programs in one session. Students assigned to the experimental group participated in a 2-h training session. Students randomised to the lecture group attended a 1-h lecture with the same clinical cases as the experimental group. Then they attended outpatient clinic for 1 h after the lecture. Students assigned to the no intervention group attended outpatient clinic for 2 h (Fig. 1). All students were informed that the test scores in this study were excluded from the final grades. Blinding was not feasible because of the different nature of interventions.

This study was exempted from full review by the Institutional Review Board, and the requirement for obtaining informed consent from the participants was waived by the board, because this study aimed to investigate the effectiveness of instructional techniques in established educational settings. Bioethics and Safety Act in Korea stipulated that such research can be exempted. This study was registered at clinicaltrials.gov as NCT03472001.

### Intervention in the experimental group

The design goal of the experimental group was to encourage students to improve their clinical reasoning by practicing to think critically like a dermatologist, and to help students build adequate illness scripts of skin diseases. Three to four students and one instructor who was a board-certified dermatologist (H.S.Y.) participated in each training session, which was conducted for 2 h. Approximately 10 min were required to complete the following steps for each case:

1. Written clinical cases with photographs

**Fig. 2:**
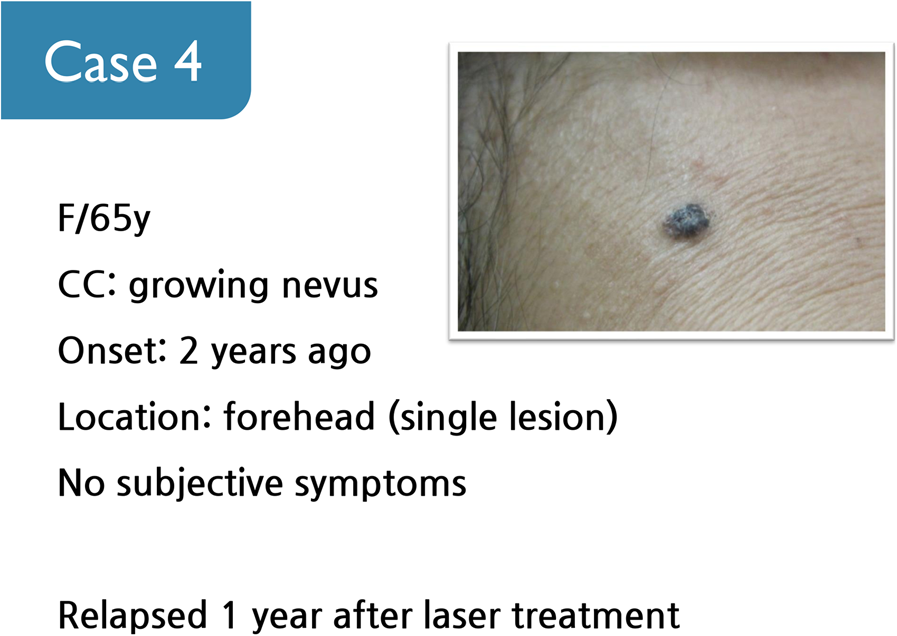
An example of a training case. This is a case of pigmented basal cell carcinoma. CC, chief complaint; F, female

This study used 10 written clinical cases for practice (training cases in Table 1). All cases were based on real patients whose diagnoses were confirmed by diagnostic work-up or treatment responses. We selected clinical cases that were encountered by non-dermatologists (e.g., primary physicians or other specialists) and had an incorrect initial diagnosis at first. Of the 10 cases, 9 were organised to enable students to compare and contrast adjacent diseases, which were different diseases that showed considerable overlap in terms of symptoms or signs. Each case consisted of a brief description of the patient’s medical history in a bulleted list and a photograph of the patient’s skin lesion (Fig. 2). Cases were presented as PowerPoint slides. Additionally, three to five essential components in each case to make a correct diagnosis were defined.

2. Abstractions in medical terminology

**Table 1:** The list of diagnoses in the control and training sets

Training cases (***N*** = 10)	Control cases (***N*** = 10)
Herpes zoster	Alopecia areata
Recurrent herpes simplex virus infection	Actinic keratosis
Allergic contact dermatitis due to ginkgo tree fruit	Seborrheic keratosis
Small plaque psoriasis	Wart
Large plaque psoriasis (confused with eczema)	Vitiligo
Bowen’s disease	Urticaria
Pigmented basal cell carcinoma	Tinea
Intradermal nevus	Varicella
Longitudinal melanonychia of a child	Pityriasis versicolor
Scabies	Infantile hemangioma

All students were requested to write down a description of the patient’s skin lesion using abstract terms called semantic qualifiers [16]. Students were able to refer to lists of semantic qualifiers of dermatologic descriptions in their textbooks and notes. Next, students verbalised what they have observed using appropriate semantic qualifiers for the case.

3. Initial diagnosis by students

Students were asked to state the most likely diagnosis for the case. Every student had to present his or her own diagnosis.

4. Correct diagnosis

The correct diagnosis of the case was shown after all students arrived at their initial diagnoses.

5. Reflection and feedback

Students were requested to reflect on the case. They were asked to list findings in the case that were essential to making the correct diagnosis. Next, they had to discuss their thoughts, one after another, using appropriate semantic qualifiers. The instructor provided immediate feedback following each student’s presentation. When student’s thought was concordant with one of the predefined diagnostic components, the instructor confirmed whether the student’s thought was relevant. When student’s thought differed considerably from the predefined components, the instructor explained why it was not essential for the diagnosis (e.g., when one student presented that old age was essential to the diagnosis of herpes zoster, the instructor responded that paediatric patients are commonly encountered, even though herpes zoster is prevalent in elderly patients). If students had difficulty identifying relevant findings, the instructor would give a hint or a cue by asking a question (e.g., “Can a diagnosis be changed if multiple lesions are present?).

6. Further evaluation (optional)

In some cases, students were asked to analyse findings that should have been present in certain skin conditions or laboratory tests that they should perform to confirm the diagnosis (e.g., skin biopsy).

### Intervention in the lecture group

We had already conducted the same training course for the experimental group in 2017 and obtained students’ common answers and misunderstandings. During the lecture, the instructor explained the findings, including why the diagnosis was drawn and how to differentiate it from other diagnoses, acquired through reflection and feedback by the experimental group. The intervention in the lecture group used clinical cases identical with those of the experimental group. The lecture was delivered as a video (PowerPoint slides with a narration) provided by the same instructor of the experimental group to maintain quality. To encourage students’ concentration, a chief resident attended the lecture session as a supervisor while the video was playing.

### Outcome

All students took a test before (baseline test) and after completing the 2-week rotation (final test). Students administered the baseline and final tests at the first and last day of each rotation, respectively. They diagnosed 10 novel cases (i.e., the patients were different than those in the training session) of diseases that were also presented in the training session (training set), and 10 cases of new diseases that were not included in the training session (control set in Table 1).

During the test, students were requested to read each case and write down the two most likely diagnoses for the case. When the answer was correct, either of the two diagnoses, students got one point. The list of diseases of the two tests were the same; however, the patients were different in the baseline and final tests. Students did not know the correct answers not until after the tests.

### Statistical analysis

We enrolled all students participating in the dermatology electives in 2018, instead of calculating the sample size. Descriptive results were expressed as means±standard deviations (SDs). A linear mixed effects model was used to compare the baseline and final test scores among the three groups (diagnostic accuracy) and within group from the same subjects. The model included intervention, set (training vs. control), time point (baseline vs. at the end of rotation), and all of their possible interaction terms. Intervention, time, and set were included as fixed factors, and a random intercept as a random factor was considered in the linear mixed effects model. Our model was assessed by the Bayesian information criterion [17]. Furthermore, differences between the means of the three groups were determined by Tukey’s post hoc pairwise test for multiple comparisons.

The effect size of intervention was calculated as Cohen’s d with values of 0.2, 0.5, and 0.8 indicating small, medium, and large effects, respectively [18]. SPSS version 20.0 (SPSS Inc., Chicago, IL, USA) was used for the statistical analysis. *P* < 0.05 was considered statistically significant.

## Results

A total of 87 students underwent randomisation (male 50, female 37). There was no significant difference in sex distribution among the three groups (*P* = 0.965 by the chi-square test) supporting that the randomisation was appropriate and the three groups were comparable.

The three-way linear mixed effects model showed significant main effects of the intervention and time on the test score (diagnostic accuracy), whereas set did not achieve statistical significance at this level. However, there was a significant interaction effect of intervention×set×time (Table 2). A post hoc analysis indicated that the students in the experimental group outperformed students in other two groups only in the training set of the final tests; after completing the 2-week rotation, the mean score for the training in the experimental group (7.5 ± 1.3) was higher than that in the lecture (5.7 ± 1.6) and no intervention (5.6 ± 1.3) groups (Table 3). The experimental group showed a large effect size of 1.5 SD vs. the no intervention group and 1.2 SD vs. the lecture group. For the control set, there were no differences in the final test scores among the three groups (Table 3).

**Table 2:** Results of the three-way linear mixed effect model

Parameter	NumDF	DenDF	F-statistic	*P*-value
***Fixed effects***				
Intervention	2	84	5.195	0.007
Time	1	252	135.585	< 0.001
Set (training vs. control)	1	252	0.203	0.652
Intervention×time	2	252	2.621	0.075
Intervention×set	2	252	5.961	0.003
Set×time	1	252	18.398	< 0.001
Intervention×set×time	2	252	3.254	0.040
Parameter	Estimate	Standard error		
***Random effects***				
Residual	1.787	0.159		
Variance random intercept	0.604	0.167

**Table 3:** Mean scores of students in the experimental, lecture, and no intervention groups*

	No intervention	Lecture	Experiment	***P***-value^†^
	(*n* = 34)	(*n* = 26)	(*n* = 27)	
Baseline				
*Control set*	4.5 ± 1.6	4.5 ± 1.7	4.6 ± 1.6	0.974
*Training set*	4.7 ± 1.2	5.4 ± 1.8	5.6 ± 1.6	0.061
After a 2-week dermatology elective course				
*Control set*	6.4 ± 1.6	7.2 ± 1.8	6.9 ± 1.4	0.137
*Training set*	5.6 ± 1.3^A^	5.7 ± 1.6^A^	7.5 ± 1.3^B^	< 0.001

^*^Values are means ± SDs

^†^*P* values by a linear mixed effects model. Groups not sharing a common superscript (capital A, B) significantly differ in the Tukey’s post hoc test

**Fig. 3:**
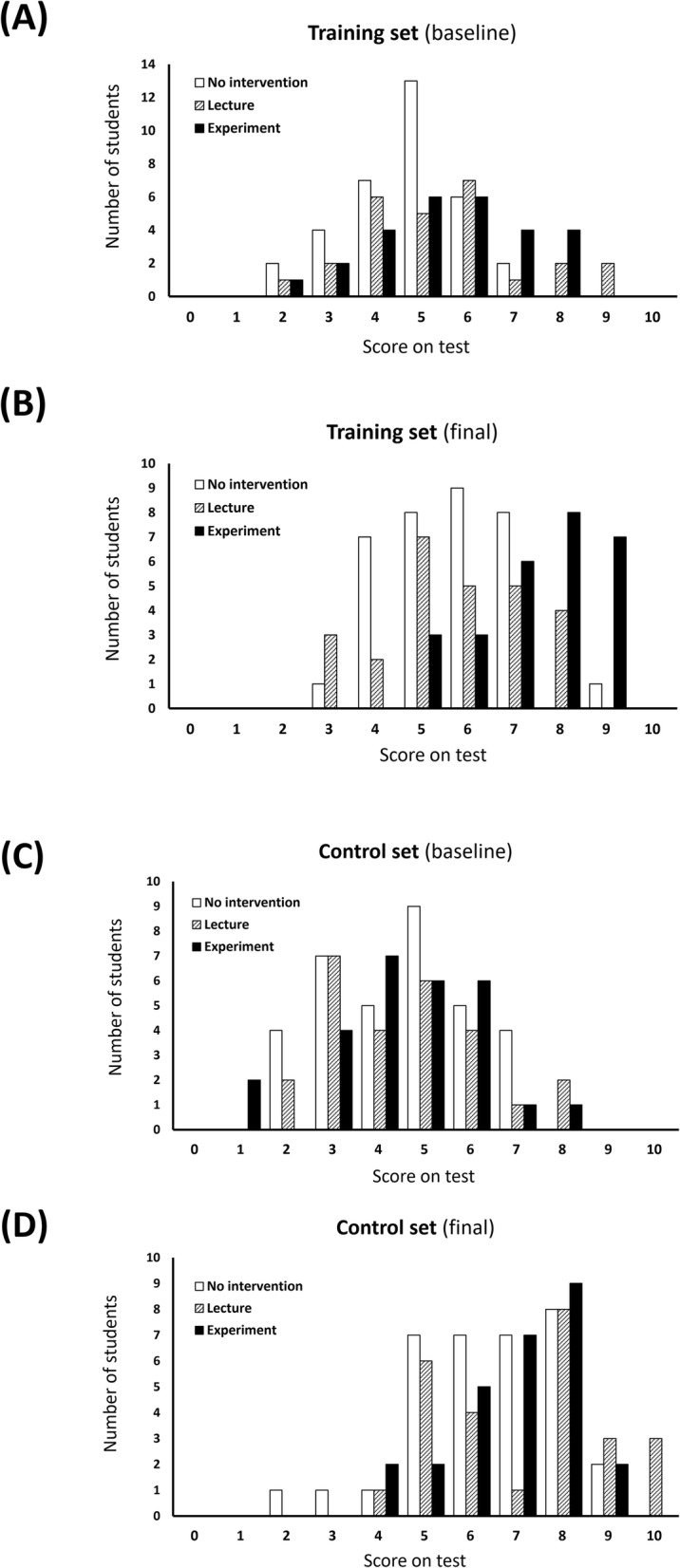
Histogram of student scores for the training (**a**, **b**) and the control (**c**, **d**) sets

The distribution of the test results is shown in Fig. 3. For the training set, the distributions of the final scores had a little overlap between the experimental and the other two groups. More than half of the students in the experimental group (15/27) were clustered in the higher scores (≥8 points) and no student obtained < 5 points, which were frequently observed in the other two groups (Fig. 3a and b). These findings demonstrate that differences in learning of the experimental group existed for essentially the entire student population. For the control set, however, all three groups showed a similar distribution and improvement after 2 weeks (Fig. 3c and d). These findings indicate that outperformance of students in the experimental group was due to the educational intervention, rather than due to baseline differences among the students in clinical reasoning.

## Discussion

This study validated whether additional practice of written clinical cases with reflection and immediate feedback (experimental group) in traditional dermatology electives could improve the ability of medical students to evaluate skin lesions, compared with a didactic lecture-based approach (lecture group) and dermatology elective alone (no intervention group). We found that our educational intervention selectively increased the diagnostic accuracy among students enrolled in dermatology electives for the training set only. The effect size was large, 1.2 SD and 1.5 SD compared with the lecture and no intervention groups, respectively.

Most studies on medical education compared the efficacy between an educational intervention and without an intervention. However, effect sizes tend to be overestimated under these conditions [19]. Our results suggested that practicing written clinical cases with reflection and feedback yielded additional benefits to a traditional dermatology elective. A dermatology elective is the most effective way to improve diagnostic accuracy in skin lesions according to a meta-analysis [15]. However, dermatology electives are not available for all medical students. For example, only half of the medical students at SNUCM can attend dermatology electives. Furthermore, students’ abilities after electives often remain unsatisfactory [20]. A study in the UK showed that medical students were not able to identify 67% of common skin lesions after completing dermatology electives, even though the overall diagnostic accuracy substantially improved from 11 to 33% [8]. Therefore, more effective educational strategies to improve clinical reasoning in dermatology are needed, even for those who have a chance to attend dermatology electives.

In this study, our experimental intervention improved scores for the training set only. In other words, students in the experimental group appeared to only develop clinical reasoning skills of the diseases they had practiced in a training session. These findings are consistent with the belief that clinical reasoning skills are developed based on a disease-specific manner, not by improving general clinical reasoning ability [10].

We did not find any additional effect of didactic lecture compared with dermatology elective alone (no intervention group). The clinical cases in the lecture group was identical with those of the experimental group, and the actual duration of educational intervention was not different between the groups (2 h). In this study, students attended outpatient clinic while they did not receive extra educational intervention. Therefore, the difference between the groups originated from the difference in educational interventions.

Lecture (didactic approach) is the most frequent scope of educational practice to enhance participants’ abilities to diagnose skin lesions, and it has a medium effect size compared with no intervention [15]. For the control set, we also found that the final scores of the students improved compared with the baseline scores. However, our results showed that the students in the lecture group did not achieve a better diagnostic accuracy compared with the students in the no intervention group. These data could be partly explained by the following: (1) students assigned to the no intervention group actually received educational intervention, because all students in this study attended dermatology electives; and (2) all students had already taken 10-h dermatology lectures before attending the electives. Thus, they already had basic knowledge on dermatology.

In medicine, reflection is defined as the consideration of the larger context, the meaning, and the implications of an experience or action [14]. However, in clinical teaching, it is probably employed less frequently than it should be, although it is essential in educating physicians in order to help them build illness scripts [14]. To develop diagnostic competence, students must construct their own illness script based on the patient care that they have experienced because illness scripts cannot be transmitted directly from teachers to students [6, 21]. However, experience alone is not sufficient for this process. The experience must be interpreted and integrated into existing knowledge structures to become new or expanded knowledge for the students, through reflection [22]. The role of clinical teachers is to facilitate students’ reflection, and for this to be effective it requires both support and challenge [22].

In this study, we used reflection, wherein students were encouraged to reflect on the case findings that were essential to reaching the correct diagnosis. The clinical teacher should point out diagnostically meaningful information in the data of the case, identify redundant or irrelevant findings, and highlight the discriminating features, including their relative weight or importance for drawing conclusions about the correct diagnosis [12]. Some of the students assigned to the other groups (no intervention or lecture groups) could use reflection depending on their preference. In contrast, all students in the experimental group was forced to reflect on every case.

Reflection enables students to improve their clinical reasoning skills, even though they do not receive feedback [6]. However, feedback is necessary for an effective reflection [22]. Feedback is defined as specific information regarding the comparison between a trainee’s observed performance and a standard, given with the intent of improving the trainee’s performance [23]. Feedback has a beneficial effect on clinical reasoning [12, 13] and skill acquisition [24]. Unlike physical skills that an instructor can directly observe, it is difficult to give appropriate feedback about students’ mental processes, such as clinical reasoning, without knowing their thoughts. Therefore, we asked students to verbalise their thoughts instead of thinking internally, so that they may receive relevant feedback that would help them to refine their thought processes.

There are two types of feedback, which are as follows: process-oriented and outcome-oriented feedback [25]. The type of feedback used in this study was process-oriented. The goal of process-oriented feedback is to provide new instruction about how to reach a standard, rather than informing the student solely about correctness [26]. In contrast, outcome-oriented feedback provides performance-specific information (i.e., information that focuses solely on correctness) [27]. Different types of feedback can have varying influences on learning. Research regarding the training of medical students has supported the beneficial effects of process-oriented feedback compared with outcome-oriented feedback [26, 27]. For example, process-oriented feedback had a more positive influence on medical students’ efficiency in learning laparoscopic knot-tying compared with outcome-oriented feedback [26].

This study had some limitations. First, we simply chose the sample size based on realistic reasons (the number of students participating dermatology electives) instead of calculating the sample size to obtain enough statistical power. Potential difference in students’ experiences during the periods was inevitable and might influence the results. However, the control of students’ experiences throughout the electives was not feasible. To distribute potential confounders across groups, we designed a randomized controlled trial. However, regardless of random allocation, our sample size might not be big enough to prevent this problem completely.

Second, diagnostic difficulty in control and training sets might be different. Diseases of the control set were relatively easier to diagnose than those of the training set. Diseases in the control set are generally diagnosed through morphologic characteristics. In this study, final scores for the control set improved without inter-group differences. By contrast, diseases in the training set usually require additional information to morphologic characteristics. Actually, cases in the training set were misdiagnosed by non-dermatologists at first. Nevertheless, the key finding that only students in the experimental group showed improvement in the training set is still valid.

Lastly, we did not examine the long-term effects of the intervention. One study evaluating the effectiveness of dermatology electives showed that half of the initial effects disappeared after 12 months [8]. Only a limited number of students receive dermatology education after completing the dermatology electives; thus, further study is needed to evaluate the long-term effects of our educational intervention.

## Conclusions

We found that practicing written clinical cases with reflection and immediate feedback yielded additional benefits to a traditional dermatology elective. This study implies several possibilities. The educational intervention we used can be easily applied to the current medical training system because it does not require any special facilities. Medical students do not encounter a sufficient number of relevant clinical cases during dermatology rotations [8]; thus, our intervention is a good method that could be used to supplement a traditional dermatology elective. Additionally, in the era of computer science, algorithm-assisted feedback adopting our strategies can be developed and help medical students or primary physicians to achieve a higher level of clinical competence for the era of AI.

## Data Availability

The datasets used and analyzed during the current study are available from the corresponding author (HSY) on reasonable request.

## Abbreviations

ANOVA: Analysis of variance
CI: Confidence interval
SD: Standard deviation
SMG-SNU: Seoul Metropolitan Government-Seoul National University
SNUCM: Seoul National University College of Medicine
